# Advanced Temperature Design for Dynamic Performance Enhancement of PEMFCs Under High Current Density (HCD)

**DOI:** 10.1002/advs.202501825

**Published:** 2025-04-25

**Authors:** Fengyang Cai, Shanshan Cai, Zhengkai Tu, Siew Hwa Chan

**Affiliations:** ^1^ School of Energy and Power Engineering Huazhong University of Science and Technology Wuhan 430074 China; ^2^ Energy Research Institute Nanyang Technological University 50 Nanyang Avenue Singapore 637553 Singapore

**Keywords:** dynamic response characteristic, heat management, high current density, proton exchange membrane fuel cell, temperature gradient

## Abstract

The dynamic performance of proton exchange membrane fuel cells (PEMFCs) under high current density (HCD) rapid loading is crucial for commercialization. This study introduces an advanced temperature difference (TD) design featuring an in‐plane temperature gradient. By reconstructing cooling channels, optimal temperature distribution across the upstream, midstream, and downstream regions achieves balanced water‐gas‐heat conditions, enhancing the dynamic response of PEMFCs under HCD loading. Various TD designs are investigated across a broad humidity range, innovatively focusing on key moments involving load initiation, transient voltage minimum (TVM), and steady‐state voltage (SSV). Comprehensive evaluations encompassing voltage response and energy consumption assess TD enhancements, while electrochemical impedance spectroscopy (EIS) and local current density monitoring further elucidate underlying mechanisms. Results show the positive temperature difference (PTD) design enhances hydration upstream and mitigates flooding downstream under low‐humidity conditions. Conversely, the negative temperature difference (NTD) design tends to dehydration upstream and flooding downstream. At RH = 35%, the PTD design increases TVM by 18.2%, decreases voltage undershoot (VU) by 12.5%, raises SSV by 5.67%, and enhances electricity output by 7%. As humidity increases, the positive effect of the PTD design gradually weakens, though it still benefits the current density distribution uniformity.

## Graphical abstract

This study presents an innovative temperature difference (TD) design with an in‐plane gradient to optimize cooling channels in proton exchange membrane fuel cells (PEMFCs) under high current density (HCD) loading. The positive temperature difference (PTD) design improves dynamic response by enhancing hydration upstream and mitigating flooding downstream, showing a 7% increase in electricity output at low humidity.

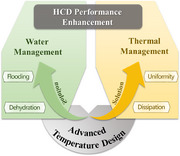

## 1 Introduction

Hydrogen energy, recognized for its clean and sustainable alternative to fossil fuels, has increasingly been recognized as a pivotal solution for the global energy transition.^[^ ^1^, ^2^, ^3^ ^]^ Proton exchange membrane fuel cells (PEMFCs), a key technology for electricity generation in the hydrogen era, have emerged as a critical solution, distinguished by their high efficiency and environmental benefits.^[^ ^4^ ^]^ They are widely applied in sectors such as electric vehicles and portable electronics. The electrochemical reaction between hydrogen and oxygen in a PEMFC generates electricity, with water and heat as the only by‐products, offering the advantages of zero emissions and low noise. These attributes position PEMFCs as a promising alternative to conventional energy systems, particularly in transportation applications such as fuel cell vehicles, where their potential for widespread adoption continues to expand. The rapid development of hydrogen energy technologies has led to the gradual expansion of PEMFC applications from portable devices and light‐duty vehicles to broader scenarios, including electric heavy‐duty vehicles, stationary power generation facilities, and industrial applications.^[^ ^5^, ^6^ ^]^ Achieving stable performance at high current densities (HCDs) is a critical challenge for PEMFCs in satisfying the demand for efficient and stable power output in these applications. Particularly in the transportation sector, achieving and enhancing HCD performance is crucial for reducing system size and cost while improving overall performance, which has emerged as a widespread core objective in promoting the development of fuel cells and plays a pivotal role in global hydrogen energy strategies.^[^ ^7^ ^]^

However, pursuing HCDs presents significant challenges for fuel cells, particularly in managing thermal regulation, water balance, and mass transport.^[^ ^8^, ^9^, ^10^ ^]^ As current density increases, heat generation within the cell escalates significantly, while water production and transport under HCD conditions become progressively more challenging, with the thermal and water management issues intensifying and amplifying each other.^[^ ^11^, ^12^, ^13^, ^14^ ^]^ Effectively managing production water to prevent localized water accumulation or dryness, ensuring an adequate supply of reactant gases, and optimal conditions in the reaction zone are critical factors for enhancing the efficiency of HCD operation. Moreover, the rapid load transition to HCD imposes significantly increased demands on fuel cell performance. The response during fast load to HCDs denotes the fuel cell capacity to swiftly accommodate a sudden surge in power demand over an exceedingly brief interval, fulfilling the requirements of applications necessitating immediate high currents, including scenarios such as vehicular rapid acceleration. This rapid response is typically accompanied by instantaneous voltage fluctuations and changes in the internal water content issues, affecting both electrochemical reaction performance and potentially accelerating cell degradation.^[^ ^15^ ^]^ Therefore, enhancing the dynamic response performance of the cell by optimizing its state and reaction conditions during rapid loading has become a significant challenge.

Current research primarily concentrates on the dynamic response under rapid loading conditions at moderate to low current densities. Hamelin et al.^[^ ^16^ ^]^ were the first to investigate the transient voltage fluctuations in PEMFCs during rapid load changes, pioneering research on the dynamic performance of PEMFCs in response to varying load conditions. The response voltage initially exhibits undershoots before gradually recovering under rapid loading conditions, forming a distinct valley shape in the voltage‐time curve. The voltage undershoots responsible for this valley significantly compromises the stability and energy efficiency of the cell during the loading process. This phenomenon can be attributed to factors including the delay in reactant gas supply, non‐uniform distribution of reactants, and mass transport obstruction.^[^ ^15^ ^]^ Yan et al.^[^ ^17^ ^]^ demonstrate an increase in operating pressure contributes to enhancing the dynamic response performance of PEMFCs during rapid loading. Kim et al.^[^ ^18^ ^]^ further investigated the factors involving stoichiometric ratio and humidity of cathode reactant gas on the transient response of PEMFCs. They observed that the undershoot was more pronounced at lower humidity levels and determined the optimal air stoichiometric ratio to be between 2.0 and 2.5. Liu et al.^[^ ^19^ ^]^ effectively reduced voltage undershoot during load cycling by introducing cathode recirculation in dead‐ended cells, which mitigated mass transport limitations caused by gas starvation. The findings indicate that significant mass transport issues tend to occur due to inadequate gas supply and flooding during rapid load cycling conditions. Yu et al.^[^ ^20^ ^]^ and Ma et al.^[^ ^21^ ^]^ investigated the mechanisms of oxygen starvation and hydrogen starvation in PEMFCs using local current distribution measurement techniques, respectively. Yin et al.^[^ ^22^, ^23^ ^]^ examined the reverse current generated instantaneously during PEMFC startup, highlighting significant corrosion at the anode outlet and underlining the critical need to mitigate hydrogen starvation. Xia et al.^[^ ^24^ ^]^ investigated the impact of multi‐stage load reduction measures on mitigating the flooding impact in fuel cells. Fan et al.^[^ ^25^ ^]^ proposed that an elevated distribution ratio of Pt on the PEM side of the catalyst layer can effectively mitigate voltage undershooting during rapid load transitions while compromising the local current density distribution consistency. Yang et al.^[^ ^26^ ^]^ demonstrated superior dynamic response characteristics during load cycling for counterflow compared to co‐flow through simulations. Wang et al.^[^ ^27^ ^]^ introduced a deep learning framework utilizing Long Short‐Term Memory (LSTM), which significantly reduces computational load and effectively enhances the efficiency of PEMFC dynamic performance prediction. This approach offers a novel perspective for optimizing the dynamic response performance of fuel cells.

Cho et al.^[^ ^28^ ^]^ further conducted a quantitative analysis of the voltage dynamic response in PEMFCs during rapid load variations by defining parameters such as FTD, STD, and VU based on the voltage response characteristics and further accurately described the voltage response during load variation by quantifying these parameters. Numerous researchers have conducted extensive studies on these parameters, systematically elucidating the key factors affecting parameter performance. According to the experimental results from Chen et al.,^[^ ^15^ ^]^ the response time is predominantly influenced by the load current magnitude and stoichiometric ratio. Meanwhile, the voltage undershoot (VU) is primarily determined by the supply gas relative humidity (RH), stoichiometric ratio, and the load current magnitude. Yang et al.^[^ ^29^ ^]^ show that reducing reactant consumption rate and increasing their initial concentration enhances response stability under load variation. Notably, augmenting the initial hydrogen concentration exerts a more pronounced effect on load performance than enhancing the initial oxygen concentration. Luo et al.^[^ ^30^ ^]^ conducted an in‐depth analysis of the response process during load variations from an energy standpoint, concluding that the augmentation of load magnitude and the insufficiency of reactant supply during rapid loading are primary contributors to enhanced energy waste. Li et al.^[^ ^31^ ^]^ investigated the effect of channel design on the rapid load response performance, with results indicating that parallel flow fields demonstrate superior transient response capabilities during load changes relative to serpentine flow fields.

In addition to investigating voltage response characteristics, several studies have delved into the heat and humidity distribution within the cell during rapid loading. Kim et al.^[^ ^32^ ^]^ investigated the dynamic changes in temperature and water distribution within the cell during rapid loading through real‐time monitoring. They revealed significant fluctuations in temperature and water distribution occur at the moment of loading, which critically influence the performance and stability of the cell. Yuan et al.^[^ ^33^ ^]^ investigated the polarization phenomena within PEMFCs during transient responses and discovered a significant relationship between voltage fluctuations, the hydration state, and oxygen transport within the cell. It is particularly noteworthy that insufficient humidification can result in localized oxygen supply issues. Such cathode oxygen starvation significantly reduces the cell lifespan, primarily by damaging the catalyst layer,^[^ ^34^ ^]^ mainly through the agglomeration of platinum catalyst particles, which is more pronounced at the outlet of the cathode.^[^ ^35^ ^]^ Yang et al.^[^ ^36^ ^]^ systematically studied the water‐gas transfer characteristics during the dynamic response of dead‐end PEMFCs in the purging stage, combining simulation and experimental approaches. The findings revealed that water‐gas transfer substantially affects fuel cell performance, and optimal system control parameters were determined to ensure both the stability of the dynamic response and the efficiency of system operation.

Existing research on the dynamic response performance of PEMFCs primarily focuses on the low to medium current density range. The current demand for fuel cell electric vehicles (FCEVs) increases significantly during startup, acceleration, and high‐load operation.^[^ ^37^ ^]^ Particularly during rapid acceleration, the fuel cell must supply a substantial amount of current in an extremely short time to efficiently drive the motor. However, research and optimization of dynamic response performance during rapid loading under HCD require further enhancement. The response to rapid HCD loading gives rise to pronounced issues, such as instantaneous voltage fluctuations and moisture variations within the cell. Additionally, current studies on dynamic response performance primarily focus on voltage response, with relatively limited research on the evolution of local current density distribution during the response process. Temperature difference (TD) design is a promising strategy for enhancing the PEMFC HCD performance, particularly in addressing uneven water‐gas distribution during rapid HCD loading response. This study introduces an innovative reconfiguration of the PEMFC liquid cooling channels, constructing three relatively independent cooling channels (upstream, midstream, and downstream) along the gas flow direction. It enables temperature optimization for each region based on its specific water‐gas reaction characteristics. The TD magnitude between regions is adjusted to establish an in‐plane temperature gradient in the cell while ensuring the overall average temperature remains constant. This design can potentially enhance the dynamic response performance of PEMFCs by optimizing the temperature distribution. Combining electrochemical impedance spectroscopy (EIS) and real‐time local current density monitoring techniques, this study focuses on tracking the evolutions of voltage and local current density distribution during rapid loading to HCD, emphasizing three key moments: load initiation, the transient voltage minimum (TVM), and the steady state voltage (SSV). A systematic analysis of the effects of TD designs (including different directions and magnitudes) under various humidity conditions was conducted, further exploring their impact on PEMFC dynamic performance and the underlying mechanisms.

## 2 Experimental Section

### 2.1 Design of Hydrogen‐Air PEMFC Based on Temperature Difference (TD)

**Figure 1:**
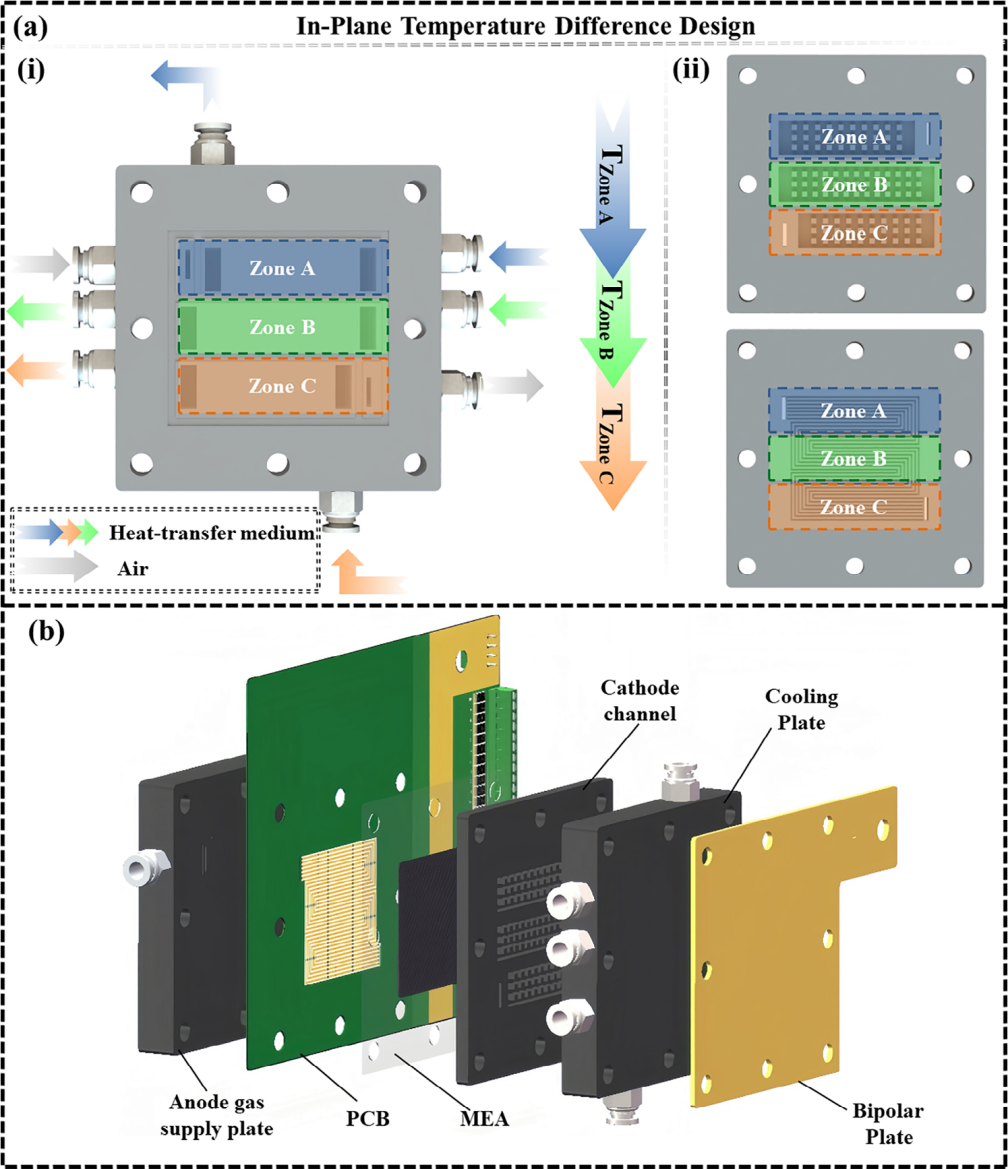
a) Conceptual structural diagram of the reconstructed cathode cooling design of the PEMFC, where (i) illustrates the cooling plate and (ii) illustrates the cathode flow field plate; b) Schematic diagram of the overall PEMFC structure with the TD design.

The structure of the hydrogen‐air PEMFC based on the TD design at the cathode is shown in **Figure** **1**. In this design, the coolant flow regions between the cooling plate and the gas flow channel have been reconfigured to ensure stable temperature control in the designated areas. A detailed view of this configuration is shown in Figure 1(a). Specifically, the cooling plate (shown in Figure 1(a)(i)) and the adjacent gas flow field plate (shown in Figure 1(a)(ii)) are each subdivided into three distinct zones: Zone A, Zone B, and Zone C, based on the in‐plane flow path of the reactant gases. The three‐zone design balances theoretical benefits and engineering practicality. It enables effective regulation of water, thermal, and gas distributions, especially at the inlet and outlet, while minimizing manufacturing complexity and cost, thereby maximizing achievable performance improvements. The separate design of each temperature zone was implemented to allow for rapid and precise control of temperature, ensuring efficient thermal management across the cell. Precisely, Zone A corresponds to the upstream section, Zone B to the midstream section, and Zone C to the downstream section of the cell. Each zone was supplied with a liquid coolant at a set temperature (deionized water was used in this study) to ensure that the cathode reactions in each zone occur at the desired temperature. An average temperature of 60 °C was maintained across the three temperature zones, with the coolant temperature in the midstream zone stabilized at 60 °C. The upstream and downstream zones exhibit identical absolute TD relative to the midstream zone while in opposite directions. To thoroughly investigate the impact and underlying factors of TD design on hydrogen‐air fuel cells under HCD conditions, this study introduces a PCB‐based flow field structure as an alternative to the standard design. This modification allows for monitoring local current density variations across different cell sections throughout the loading process, providing insights into the TD design influence during experimental operations. As illustrated in Figure 1(b), the structure from anode to cathode consists of the following components: anode gas supply plate, PCB board, MEA, cathode flow field plate, cathode cooling plate, and bipolar plate. The structure differs from traditional water‐cooled fuel cell designs by introducing an innovative approach to the cathode water‐cooling channels. As shown in Figure 1(a), three independent water‐cooling channels are evenly distributed along the in‐plane direction of the reactant gas flow, ensuring effective temperature control and achieving the desired TD design. Additionally, the traditional anode flow field plate is replaced with a PCB in this design, which was positioned between the anode gas supply plate and the MEA. By dividing the PCB into 30 independent insulated regions, the current density in each region can be monitored separately, enabling a more accurate investigation of the effect of TD on the rapid loading response performance of the fuel cell under HCD conditions and its underlying mechanisms. The specific design parameters of the fuel cell are listed in **Table** **1**. A seven‐serpentine flow field with an active area of 25 cm^2^ is used, where the flow channel width and depth are 0.6 mm and 0.5 mm, respectively, with a rib width of 0.8 mm. The gas diffusion layer and proton exchange membrane thicknesses are 0.18 mm and 12 µm, respectively.

**Table 1:** Design and operating parameters of PEMFC.

Parameter	Value	Unit
MEA active area	25	cm^2^
Channel width	0.6	mm
Channel depth	0.5	mm
Rib width	0.8	mm
Flow channel property	7‐multi‐serpentine	/
Pt loading (anode/cathode)	0.1/0.4	mg cm^−2^
GDL thickness	0.18	mm
Membrane thickness	0.012	mm

### 2.2 Description of the Experimental Setup and Testing Strategy

**Figure 2:**
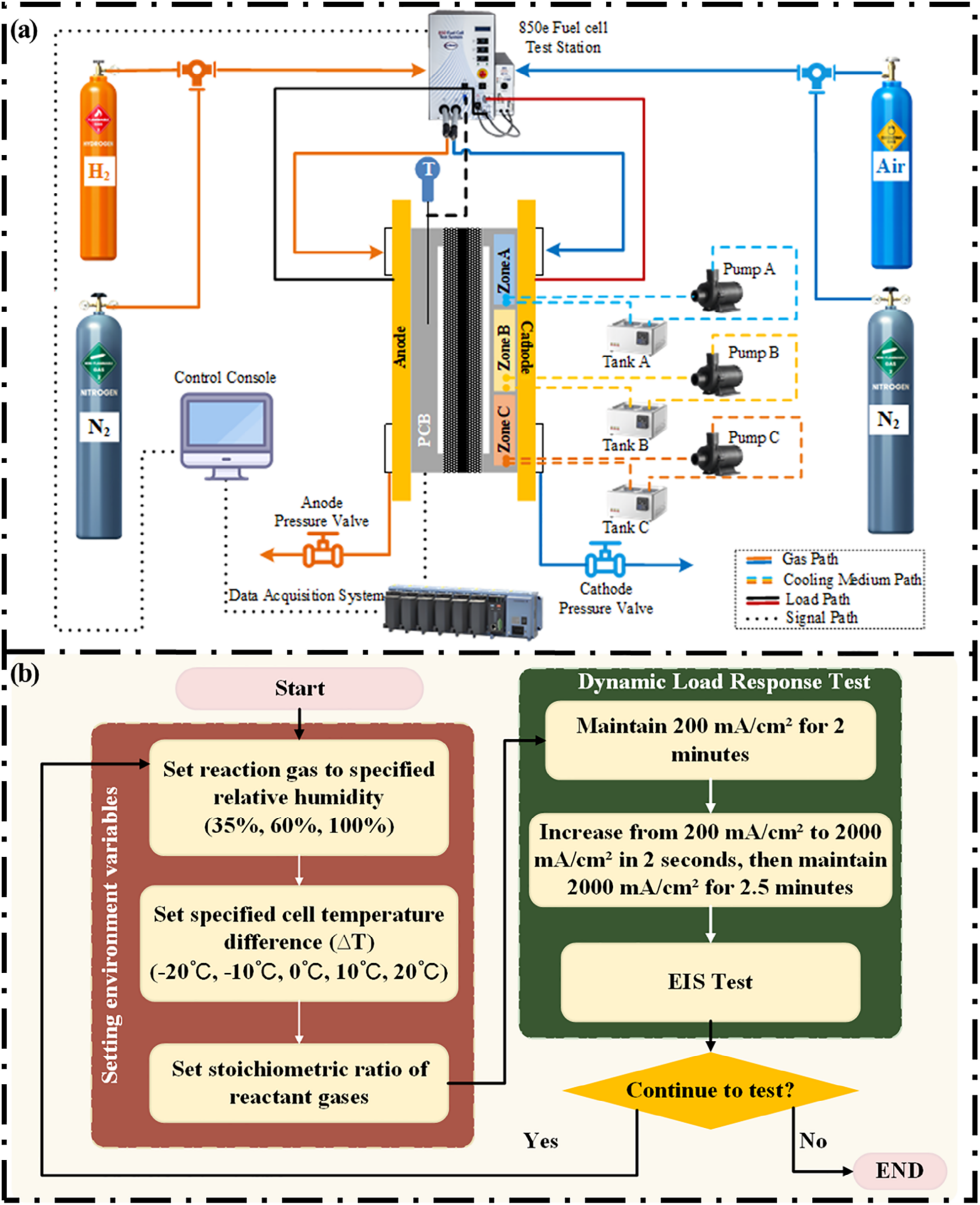
a) System architecture of the hydrogen‐air PEMFC with TD design and the corresponding b) experimental procedure.

**Figure** **2** further elucidates the system architecture and control strategy of the PEMFC with TD design during HCD load response testing. The primary configuration of the system, as illustrated in Figure 2(a), comprises a gas supply system, a single fuel cell, a control system, a data acquisition system, and a coolant management system. The gas supply system primarily comprises reaction gases for the cathode (air) and anode (hydrogen), along with purge gas (nitrogen), all of which have a purity level of at least 99.999%. The reaction and purge gases are supplied to the main control panel (850e fuel cell test station), which provides the required flow rate and RH of gases to the single cell according to preset parameters and load while controlling the anode to the required temperature. The TD distribution at the cathode of the single cell is achieved through three independent water tanks and pumps, which supply deionized water at precisely controlled temperatures. Temperature sensors embedded at the cooling plate outlets continuously monitor the water temperature to ensure it remains at the set value. The acquisition system gathers data via a centralized control terminal. Specifically, the 850e acquisition system supplies the control terminal with cell voltage and load data, and the system provided by Yokogawa GM collects real‐time local current data for each section of the cell to the control terminal. The backpressure valve can dynamically adjust the cell backpressure in response to variations in gas flow, ensuring it remains at the operator‐specified value. Figure 2(b) illustrates the flowchart of the experiment, which aims to investigate the effects of various cathode TD designs on the rapid load response performance of hydrogen‐air fuel cells under varying inlet gas humidity conditions. During the experiment, the reaction gases for the cathode and anode are air and hydrogen, with stoichiometric ratios of 2.5 and 1.5, respectively. The back pressure is maintained constant at 20 kPa, the anode temperature is fixed at 60 °C, and the cathode temperature follows the designed TD (∆T) structure. In this study, ∆T is defined as the temperature difference between two adjacent temperature zones of the cathode. The temperature distribution for each temperature difference design is illustrated explicitly in **Table** **2**. The experimental procedure involves the following steps: first, setting the inlet humidity to low (35% RH), medium (60% RH), and high (100% RH) levels; second, controlling the anode temperature and cathode temperature difference (∆T = ±20 °C, ±10 °C, 0 °C) to the desired values. The fuel cell is first operated at a low current density (200 mA cm^−2^) for 2 min (corresponding to the idle condition of the actual vehicle operation). Subsequently, the current density is rapidly increased to 2000 mA/cm^2^ within 2 s and maintained for 2.5 min to reach a steady state, followed by EIS testing at 2000 mA/cm^2^ conditions.

**Table 2:** Temperature design for various zones in the experiments.

Temperature Difference (∆T)		Designed temperature (°C)		
Value (°C)	Properties	Zone A	Zone B	Zone C
−20	Negative	80	60	40
−10		70	60	50
0	Zero	60	60	60
10	Positive	50	60	70
20		40	60	80

### 2.3 Evaluating Parameters

A suite of evaluation parameters has been established to assess the response performance of PEMFCs under rapid HCD load cycling accurately and comprehensively. Transient Voltage Minimum (TVM) is defined as the minimum voltage reached by the cell during response to rapid HCD loading. Steady State Voltage (SSV) is defined as the final stable voltage reached by the cell after rapid HCD loading, with voltage fluctuations not exceeding 1% serving as the benchmark in the experiment. Voltage Undershoot (VU) is defined as the difference between the TVM and the final SSV during the rapid HCD loading of the fuel cell. VU reflects the extent of voltage fluctuation in the cell dynamic response and is commonly employed to assess the stability of the cell performance under rapid loading conditions as defined in the equation presented below:

(1) VU=SSV−TVM

where VU, SSV, and TVM are all in volts (V).

Energy Loss (*E*_loss_) is defined as the energy waste caused by the voltage undershoot during the rapid loading of the fuel cell, which is calculated using the following formula:

(2) $$E_{loss}=E_{ideal}\;-\;E_{act}=\int_{t_{1}}^{t_{2}}U_{SSV}I_{i}dt\;-\int_{t_{1}}^{t_{2}}U_{i}I_{i}dt$$

where, *t*_1_ and *t*_2_ refers to the start loading time and the final time (s) when the SSV is reached, respectively, *E_ideal_* represents the electricity output (J) under the ideal condition that the voltage remains constant at the final SSV throughout the whole loading response process. *E_act_* refers to the actual electricity output (J) calculated based on the actual voltage changes during the loading response process. *U_i_* and *I_i_* represent the voltage (V) and current (A) at each moment, respectively, and *U_SSV_* is the voltage value of the final response SSV (V).

To quantitatively compare the electricity output during the response process under varying TD designs as opposed to the zero temperature difference (ZTD) design, the Energy Gain Ratio (EGR) is introduced. The EGR is the ratio of the extra electricity output during the response process under the designed TD condition, relative to the corresponding electricity generation under the ZTD design (∆T = 0 °C). The formula for EGR is given by:

(3) $$\mathrm{EGR}\;=\frac{E_{\mathrm{act}}^{\Delta T}-E_{\mathrm{act}}^{\Delta T=0{}^{\circ}C}}{E_{\mathrm{act}}^{\Delta T=0{}^{\circ}C}}\;\times 100\%,\Delta T=0,\pm 10,\pm 20{}^{\circ}C$$

where $E_{act}^{\Delta T}$ refers to the electricity generation for each TD design (J) and $E_{act}^{\Delta T=0^{\circ }C}$ refers to electricity output in ZTD design (J).

To more clearly illustrate the variations in the contribution rates of the upstream, midstream, and downstream regions to the overall current density during the load response, the local current density contribution rate (denoted as ξ) is introduced and expressed as:

(4) $$\xi_{Zonex}=\frac{I_{Zonex}\times s_{Zonex}}{\sum_{q=ZoneA}^{ZoneC}I_{q}\times s_{q}}\;,x=A,B,C$$

where $I_{Zonex}$, $s_{Zonex}$ and $\xi_{Zonex}$, x=A,B,C denotes the average current density (mA/cm^2^), corresponding region area (cm^2^), and contribution to the total average current density for Zone A, Zone B, and Zone C, respectively. $I_{Zonex}$ represents the local current density in the upstream, middle, and downstream regions (Zone A, B, and C, respectively), reflecting the electricity generation capacity of each area under the same current load. The specific quantification formula is as follows:

(5) $$I_{Zonex}=\frac{\sum I_{j}\times s_{j}}{\sum s_{j}}\;,x=A,B,C$$

where the subscript *j* is the actual measurement site corresponding to the calculated region.

The standard deviation of local current density, σ, is established to assess local current density uniformity under TD effects. Smaller σ values denote higher uniformity, whereas larger values indicate pronounced inhomogeneity, which is defined as:

(6) $$\sigma =\sqrt{\frac{\sum_{j=1}^{N}s_{j}{\left(I_{j}-\bar{I}\right)}^{2}}{\sum_{j=1}^{N}s_{j}}}$$

where *N* refers to the total amount of discrete areas used to assess local current density, *s* corresponds to the effective region of the MEA linked to the measured local current density (cm^2^). *I_j_* stands for the current density at a specific location (mA/cm^2^), whereas $\bar{I}$ denotes the overall average current density (mA/cm^2^).

## 3 Results and Discussion

### 3.1 Optimizing TD Design for Improved HCD Dynamic Response Under Low Humidity

**Figure 3:**
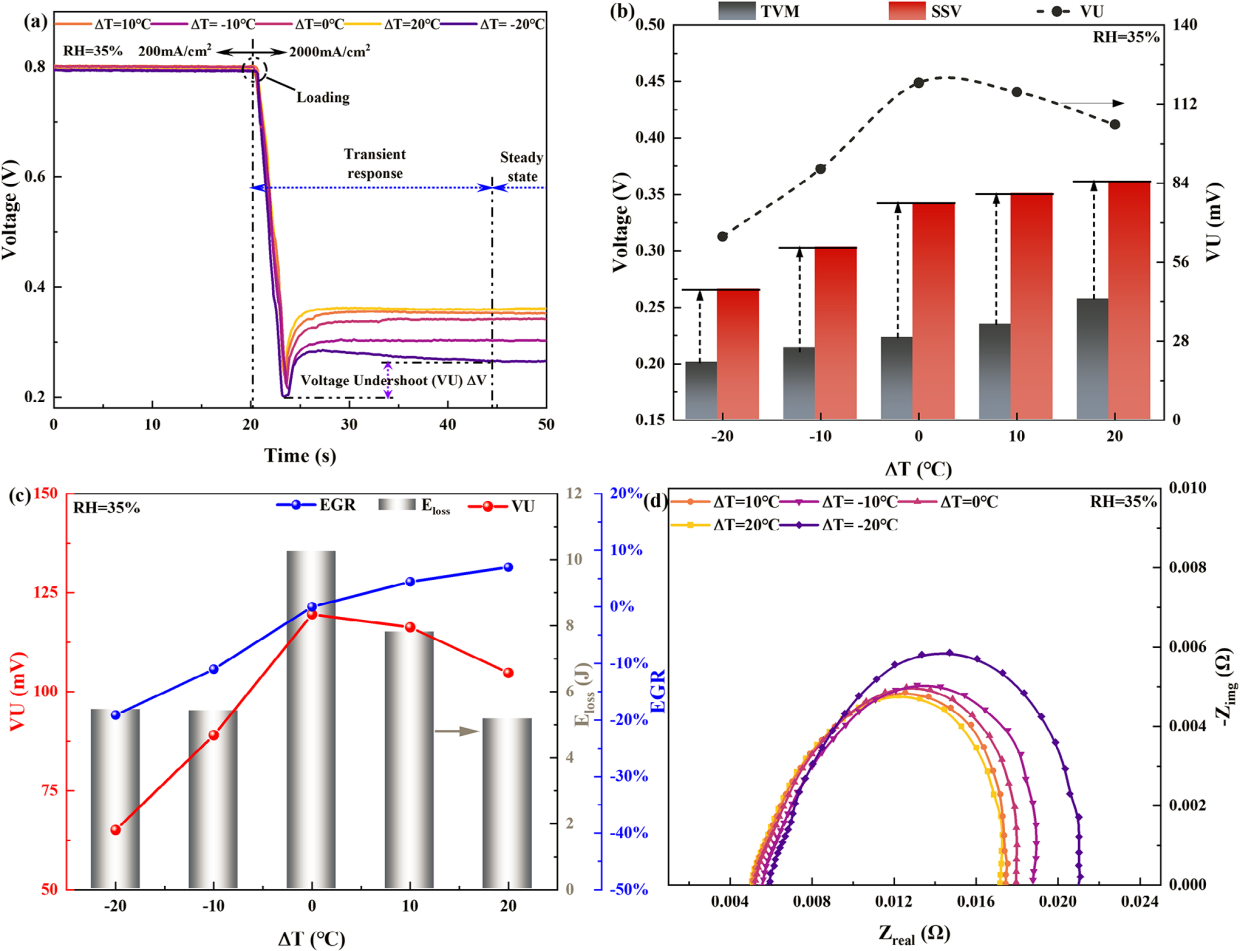
HCD rapid load response performance under different TD design conditions at RH = 35%: a) Voltage response; b) Effect on performance evaluation parameters during the dynamic loading process, including TVM, SSV, and VU; c) *E*_loss_ and EGR; d) Comparison of EIS at 2000 mA cm^−2^.

**Figure** **3** illustrates the impact of different in‐plane TD designs on the dynamic response characteristics of the cell during HCD rapid loading under low humidity conditions (RH = 35%). Significant variations in key response metrics, including TVM, VU, and SSV, are observed across the various TD designs when the load increases rapidly from 200 mA cm^−2^ to 2000 mA cm^−2^. Figure 3(a) illustrates the evolution of the voltage response comparison under different TD designs. It can be concluded that during the rapid load increase to HCD, the positive temperature difference (PTD) design demonstrates a clear advantage over the ZTD design, particularly in terms of the TVM and SSV, while the negative temperature difference (NTD) design shows a detrimental effect. The significant parameter variations during rapid load response at HCD under different TD designs, shown in Figure 3(a), are quantified and compared in Figure 3(b),(c). When the designed TD (∆T) is increased from 0 to 20 °C, TVM rises from 0.22 to 0.26 V, showing an increase of 18.2%. Within the PTD range (0 < ∆T < 20 °C), the increase in TVM becomes significantly more pronounced as ∆T increases. In contrast, when the ∆T design is set at −20 °C compared to 0 °C, TVM decreases by 9.8%, exhibiting a more pronounced voltage undershoot in response to a rapid load increase. This improvement is attributed to optimizing the temperature distribution between the upstream and downstream regions of the cell achieved through PTD design, which mitigates the reactant supply delay effects and non‐uniform distribution during rapid load increases. Specifically, the PTD design reduces the dryness in the upstream region by lowering its temperature. Although the temperature increase downstream leads to a slight decrease in local humidity, the negative effect on membrane hydration downstream remains limited due to the accumulation of reaction water and the relatively low gas flow rate downstream at the moment of the load elevation to high current. Overall, the PTD design can effectively reduce the whole ohmic resistance during rapid high current loading by optimizing the relative humidity between the upstream and downstream regions, further explained in Figure 3(d). In contrast, the NTD design, characterized by its higher upstream and lower downstream temperatures, increases overall ohmic resistance during rapid load increases. Additionally, VU decreases by 12.5% and 45.8%, respectively, when the temperature difference is ∆T = 20 and −20 °C, compared to ∆T = 0 °C. An apparent reduction of the VU is present in both PTD and NTD designs compared to the ZTD under low humidity conditions, and the decrease in VU becomes more pronounced as the absolute value of ∆T increases within the 0–20 °C range. However, the underlying mechanisms to mitigate VU differ significantly between PTD and NTD designs. For the PTD design, the reduction in VU is primarily achieved by a significant increase in TVM and a moderate improvement in SSV. In contrast, the NTD design reduces VU mainly at the expense of SSV, rendering this optimization approach practically less significant.

Figure 3(c) further examines the energy loss (*E*_loss_) and EGR during the rapid HCD load response resulting from voltage undershoot. It can be observed that the trends of *E*_loss_ and the VU parameter are similar during the rapid HCD load response. The PTD design achieves superior energy savings compared to the ZTD design by significantly mitigating voltage undershooting when responding to a rapid increase in HCD load. The EGR quantitatively compares the differences in actual electricity generation during the rapid high current density load response across various TD designs relative to the traditional ZTD design. It can be concluded that the PTD design significantly increases EGR, primarily due to the effective enhancement of SSV and TVM. Specifically, with a temperature difference of ∆T = 20 °C, EGR is improved by 7%. However, under the NTD design, the EGR decreases by 19% compared to the ZTD design, primarily due to the negative impact on SSV despite the reduction in VU. Figure 3(d) explains the effects of different TD designs from the perspective of EIS. It can be concluded that under the same average temperature, the PTD design effectively reduces the ohmic resistance during rapid HCD load response under low humidity by constructing an increased temperature distribution from the inlet to the outlet. When ∆T = 20 °C, compared to ∆T = 0 °C, the ohmic and polarization resistances are reduced by 3.4% and 5.1%, respectively. Correspondingly, under the NTD design with ∆T = −20 °C, compared to ∆T = 0 °C, the ohmic and polarization resistance increase by 13.4% and 18.6%, respectively. The effect of TD on ohmic and polarization resistance further demonstrates that the PTD design effectively improves membrane hydration by adjusting the temperature distribution under low humidity conditions, thereby enhancing HCD performance. In contrast, the NTD design exacerbates membrane dehydration under low humidity, negatively impacting the response performance.

### 3.2 Dynamic Evolution Characteristics of Local Current Density Under Different TD Designs in Low Humidity Conditions

**Figure 4:**
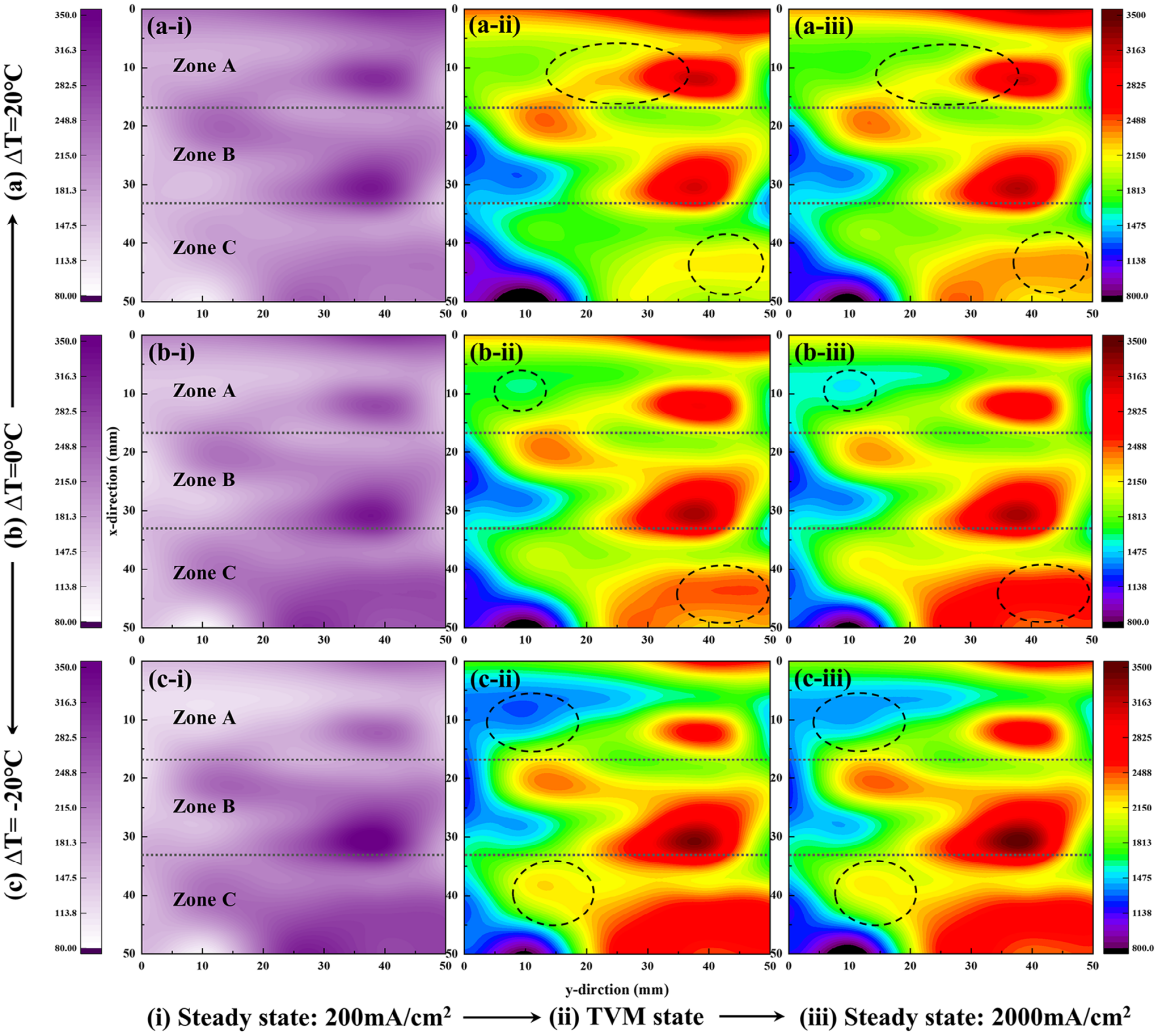
Dynamic evolution of local current density distribution during rapid HCD loading under different TD designs at low humidity conditions (RH = 35%). The TD designs correspond to ∆T = a) 20 °C, b) 0 °C, c) −20 °C. Critical moments during the response process: (i) Steady‐state at 200 mA cm^−2^; (ii) TVM state; (iii) Steady‐state at 2000 mA cm^−2^.

This section further focuses on the evolution of the local current density distribution during the rapid load increase to HCDs, providing an in‐depth investigation into the underlying principles of how TD designs influence the dynamic response during HCD fast loading of cells under low humidity conditions. **Figure** **4** illustrates the evolution of the local current density distribution of the cell under low humidity conditions during rapid HCD load increase (from 200 mA cm^−2^ to 2000 mA cm^−2^). Three typical characteristic time points are selected to represent the evolution during the response process: (i) steady state at 200 mA cm^−2^ (left in Figure 4, labeled as (i) in (a–c)), (ii) the moment of the TVM (middle in Figure 4, labeled as (ii) in (a–c)), and (iii) the moment of recovery SSV (right in Figure 4, labeled as (iii) in (a–c)). These time points are extracted and arranged sequentially from left to right, corresponding to the progression of the response. In each subfigure of Figure 4, the increase in the x‐axis values indicates the position extending from the upstream to the downstream, which has been explicitly labeled in each subfigure for clarity. To clearly illustrate the evolution pattern, key locations with representative changes in the upstream and downstream regions during the response process are highlighted with dashed circles in Figure 4. When rapidly loaded to an HCD of 2000mA/cm^2^, under ZTD (∆T = 0 °C, Figure 4(b)) and PTD (∆T > 0 °C, Figure 4(a)), the upstream performance initially increases (Zone A in Figure 4(a‐ii, b‐ii)), then gradually decreases until stabilization (Zone A in Figure 4(a‐iii, b‐iii)), while the downstream performance begins at a lower level (Zone C in Figure 4(a‐ii, b‐ii)), gradually growing to stabilize (Zone C in Figure 4(a‐iii, b‐iii)). The evolution characteristics stem from the increased supplemental gas required for the rapidly rising current demand first passes through the upstream region, resulting in a delayed gas supply, especially in the downstream area. The delay effect leads to the upstream power generation capacity increasing more rapidly than other regions, making it the primary contribution region that maintains the constant average HCD during the initial part of the response process. However, this effect diminishes over time as the downstream electricity generation capacity improves with the gradual gas supply reaching the downstream region. In contrast, under NTD (∆T < 0 °C, Figure 4(c)), the upstream performance gradually increases with no recovery, and the downstream performance shows an initial increase followed by a gradual decrease until stabilization. The excessive NTD exacerbates the already dry upstream region under low humidity conditions, which outweighs the positive effect of the higher gas flow rate, resulting in no significant advantage in upstream power generation capacity at the moment of loading. As reaction water gradually accumulates with time, the upstream performance improves, leading to a certain degree of enhancement in the power generation capacity of the upstream region during the transition from the initial response to the steady state. Similarly, excessively low downstream temperatures lead to the gradual accumulation of liquid water over time, resulting in negative flooding near the outlet when the system reaches a steady state, as reflected by the decrease in the local average current density. Both factors cause the NTD design (∆T = −20 °C) to exhibit a contrasting evolution process in the upstream and downstream regions during rapid HCD loading compared to other TD designs.

The distinct mechanisms by which each TD design influences the cell's response process are qualitatively revealed by comparing the local current density distributions at corresponding key moments across different TD designs. The PTD (∆T > 0 °C, Figure 4(a)) design increases RH in the inlet region by lowering the upstream temperature, compared to the ZTD (∆T = 0 °C, Figure 4(b)) design. As a result, this enhanced humidity significantly boosts the electricity generation capacity in the upstream area, particularly at the TVM and SSV moments, as reflected by the increased local current density during the response process. This conclusion is demonstrated in the comparison of Zone A in Figure 4, where (a‐ii, iii) is compared with (b‐ii, iii), respectively. However, in the downstream region (Zone C), electricity generation capacity is slightly reduced due to the relatively higher temperature created by the PTD, though this reduction remains minimal. In contrast, significant upstream membrane dehydration caused by the NTD design (∆T = −20 °C, Figure 4(c)), through increasing the upstream temperature, severely affects the electricity generation capacity at both the TVM and SSV moments, as illustrated by the comparison between Zone A in Figure 4(b‐ii, iii) and (c‐ii, iii). The reduced downstream temperature partially alleviates the drying issue in that region; however, flooding near the outlet occurs due to the accumulation of water generated by electrochemical reactions during the flow of reactant gases, negatively impacting the recovery of the SSV.

**Figure 5:**
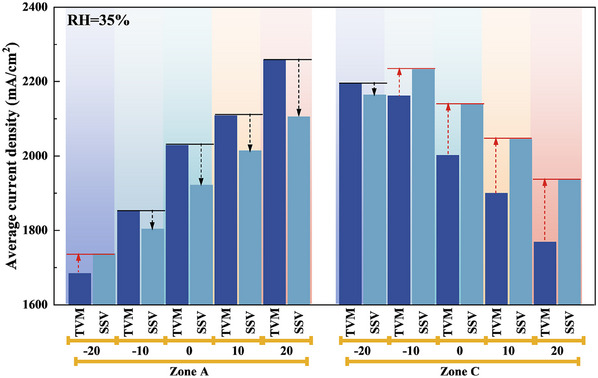
Quantitative comparison analysis of the impact of various TD designs on the local current density upstream (Zone A) and downstream (Zone C) during response to rapid loading under low humidity conditions (RH = 35%).

**Figure** **5** quantitatively investigates the evolution of local average current density in the upstream (Zone A) and downstream (Zone C) regions during rapid load increase under various TD designs in low humidity conditions, using the moments of TVM and SSV as reference points. This figure provides a more intuitive visualization of the effects of different TD designs on the response capability of the upstream and downstream regions during rapid HCD loading. For example, under ∆T = 0 °C, the upstream average current density rapidly increases, reaching 2029.32 mA cm^−2^ at the TVM moment before decreasing to a steady‐state value of 1922.26mA cm^−2^. In contrast, the downstream average current density reaches 2002.52 mA cm^−2^ at the TVM moment and gradually increases to an SSV of 2141.2 mA cm^−2^. For ∆T = −20 °C, the evolution pattern is opposite to that observed under PTD and ZTD conditions. The localized average current density evolution aligns with the conclusions from Figure 4.

Through the quantitative analysis in Figure 5, the performance impact mechanisms of the TD designs are further elucidated relative to the qualitative insights provided in Figure 4. The PTD design enhances the response performance during rapid HCD loading by improving the power generation capacity of the upstream region at both the TVM and SSV moments. When ∆T = 10 °C and 20 °C, compared to ∆T = 0 °C, the average local current density in the upstream region at the TVM moment increases by 4% and 11.4%, respectively, and the average local current density at the SSV moment rises by 4.8% and 9.5%, respectively. The positive effect intensifies with the increasing magnitude of the PTD as the difference magnitude increases within 20 °C. For the downstream region, while the PTD design increases temperature and reduces power generation at both TVM and SSV moments, the negative hydration effect is partially offset by water accumulation from reactant gases, resulting in a relatively limited overall impact on performance. In contrast, for the NTD design, the upstream region shows a decrease in average local current density at the TVM moment by 8.7% and 17%, respectively, for ∆T = −10 and −20 °C, compared to ∆T = 0 °C. Similarly, at the SSV moment, the average local current density decreases by 6.1% and 10%, respectively. Furthermore, compared to ∆T = −10 °C, the downstream average local current density at the TVM moment increased by 1.5% for ∆T = −20 °C, while at the SSV moment, the downstream average local current density decreased by 3%. The shift from positive to negative during the response period is due to the excessively high upstream and low downstream temperatures induced by the NTD design, causing upstream membrane dehydration and downstream water flooding, which worsen with increasing TD magnitude.

### 3.3 Comparison of the Impact of TD Designs on Response Performance During Rapid HCD Loading Under Different Humidity Conditions

**Figure 6:**
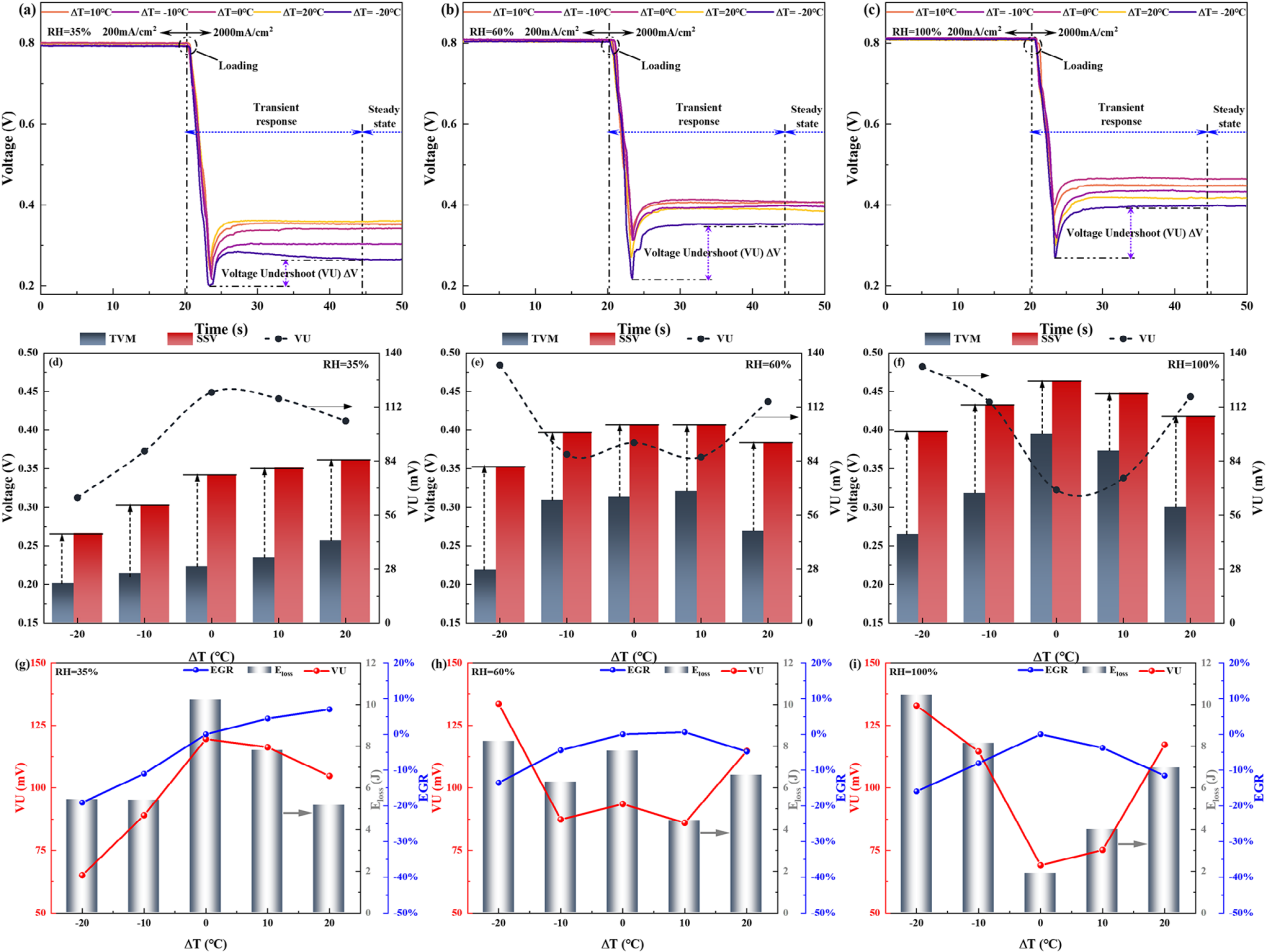
Comparison of dynamic response performance characteristics of the cell during rapid HCD loading under different humidity conditions and TD design conditions (left: RH = 35%; middle: RH = 60%; right: RH = 100%; (a–c): voltage response; (d–f): TVM, SSV, and VU; (g–i): *E*_loss_ and EGR).

The comparison of the response characteristics during rapid HCD loading for different TD designs under various humidity conditions is shown in **Figure** **6**. Figure 6 (a–c) respectively show the voltage response during the rapid load increase from low current density (200 mA cm^−2^) to high current density (2000 mA cm^−2^) under different TD designs at RH of 35%, 60%, and 100%. It can be observed that an increase in humidity improves response performance, with the enhancing effect of different TD designs varying under different humidity conditions. Overall, as the inlet humidity increases, the impact of the NTD design remains negative compared to the ZTD design, while the positive effect of the PTD design gradually diminishes. Figure 6(d–f) presents the quantitative comparison of the key evaluation parameters (TVM, SSV, VU) for voltage response during rapid HCD loading across various TD designs under conditions of gradually increasing humidity. As the RH increases from 35% to 60% and 100%, the positive effects of the PTD design on the TVM, SSV, and VU gradually decrease with both the rise in humidity and the TD magnitude. Compared to ∆T = 0 °C, ∆T = 10 °C shifts from positive to negative effects, with 5.5% and 3.4% reductions in TVM and SSV and an 8.9% increase in VU, respectively, as RH increases to 100%. In contrast, the larger TD magnitude of ∆T = 20 °C negatively exhibits 14% and 5.7% decreases in TVM and SSV and a 22.7% increase in VU at RH = 60%. The NTD design exhibits a consistent negative impact under different humidity conditions and TD magnitudes, with the negative effects on TVM and SSV becoming more pronounced as the TD magnitude increases. The overall trend of VU shows a transition from a positive parabolic shape to a “W‐shaped” curve, followed by a further transformation into an inverted parabolic shape as the intake humidity increases from RH = 35% to RH = 60% and then to RH = 100%, with the TD changing from NTD to ZTD and then to PTD. Figure 6(g–i) respectively show the evolution of EGR and *E*_loss_ during the response process under different TD designs at three progressively increasing humidity conditions. It can be concluded that *E*_loss_ follows a trend similar to VU under different TD designs, where higher humidity and TD magnitudes lead to greater energy loss. Specifically, when RH = 60%, *E*_loss_ for ∆T = 10 °C and ∆T = 20 °C decreased by 43% and 14.8%, respectively, compared to ∆T = 0 °C. However, when RH increased to 100%, *E*_loss_ increased by ≈1 and 3 times, respectively, compared to ∆T = 0 °C. EGR more clearly quantifies the advantages and disadvantages of different TD designs regarding the electricity generation during the response process relative to the ZTD design. Relative to the ZTD design (∆T = 0 °C), for the PTD design (∆T > 0 °C), the increase in electricity generation becomes more limited as the TD value grows, particularly under higher humidity conditions: When RH increases to 60%, ∆T = 10 °C only shows a 1% increase in electricity generation, while ∆T = 20 °C shows a 4.82% decrease. When RH increases to 100%, with ∆T values of 10 and 20 °C, the electricity generation decreases significantly by 3.79% and 11.58%, exhibiting a clear negative impact.

**Figure 7:**
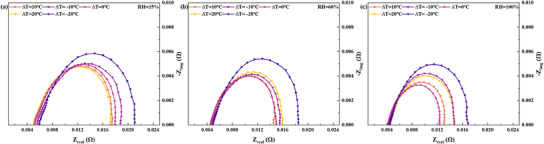
Comparison of EIS of the cell at 2000 mA cm^−2^ under different humidity conditions and TD design conditions (a): RH = 35%; b): RH = 60%; c): RH = 100%).

**Figure** **7** explains the reasons for the differences in response performance under different humidity conditions for various TD designs from the perspective of EIS. It can be concluded that as humidity increases, the impact of the ohmic resistance on the cell under HCD conditions decreases. Additionally, under the same humidity conditions, the effect of different TD designs on the ohmic resistance becomes smaller as humidity increases. Therefore, polarization resistance is the main factor influencing the HCD loading performance of different TD designs under higher humidity conditions. For the PTD design, when RH = 35%, with ∆T values of 10 and 20 °C, the polarization resistance decreases by 2.6% and 5.1%, respectively, compared to ∆T = 0 °C. When RH = 60%, the polarization resistance decreases by 4.5% and increases by 7.6% under the same conditions. At RH = 100%, with ∆T values of 10 and 20 °C, the polarization resistance negatively increases by 6.9% and 23.5%, respectively, compared to ∆T = 0 °C. It can be concluded that the positive impact of larger PTD magnitudes on HCD performance diminishes progressively as humidity increases. For the NTD design, compared to ∆T = 0 °C, the polarization resistance increases by 18.6%, 30.8%, and 51.1% at ∆T = −20 °C under relative humidity conditions of 35%, 60%, and 100%, respectively. The NTD design exerts an entirely negative effect on polarization resistance, and this impact intensifies with increasing humidity and TD magnitude.

**Figure 8:**
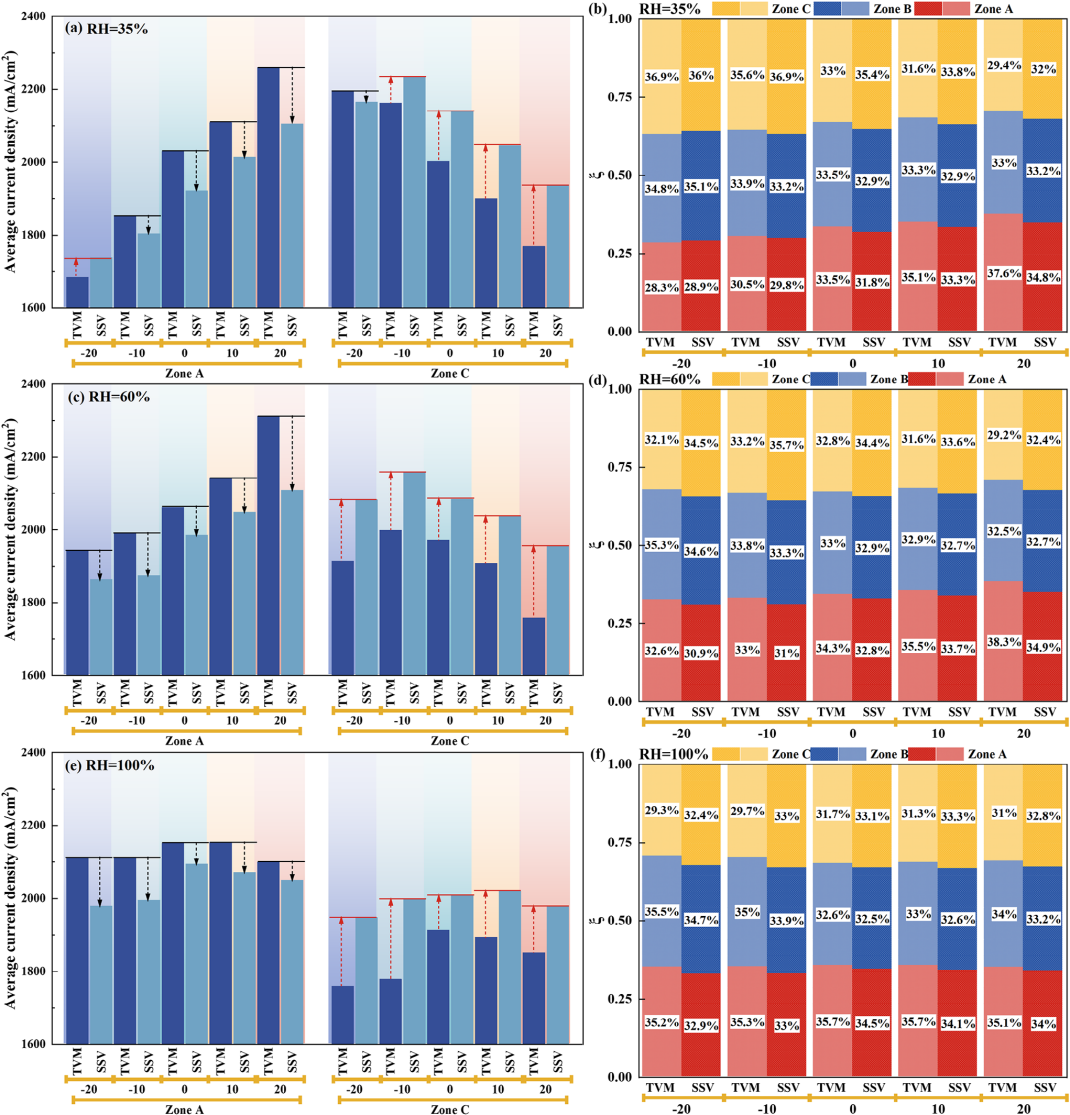
Quantitative comparison of the impact of different TD designs on the evolution of average local current density a), c), e) and contribution rate ξ b), d), f) in the upstream region (Zone A) and downstream region (Zone C) during rapid HCD loading response process under different humidity conditions.

**Figure** **8** further quantitatively compares the evolution of the local average current density and the contribution rate ξ in the upstream (Zone A) and downstream (Zone C) regions of the cell during rapid loading response under different humidity and TD design conditions. Taking the TVM and SSV moments as reference points, Figure 8 aims to explore the reasons behind the effects of different TD designs on the dynamic response performance during rapid loading, focusing on the upstream and downstream regions under various humidity conditions. For the ZTD design, when RH increases from 35% to 100%, the average local current density upstream continuously increases, with the performance contribution of the upstream region at the TVM and SSV moments rising by 6.6% and 8.5%, respectively. The increase in humidity effectively alleviates the membrane dehydration caused by ohmic resistance, enhancing the localized electrochemical reaction conditions and power generation capacity upstream, thereby increasing the local current density. Therefore, under higher intake humidity conditions, the positive effect of the PTD design on the upstream region gradually diminishes, as lower upstream temperatures may cause flooding and inhibit HCD performance. For the PTD design, as RH increases from 35% to 100%, the positive effect of the PTD on the average local current density in the upstream region gradually diminishes. Taking ∆T = 20 °C as an example, compared to ∆T = 0 °C, the contribution rate of the upstream current density increases by 12.2% at the TVM moment and 9.7% at the SSV moment when RH = 35%. This ξ enhancement (compared to ∆T = 0 °C) gradually decreases to 12% at the TVM moment and 6.5% at the SSV moment when RH = 60%, and eventually, at RH = 100%, the enhancement is −1.6% at the TVM moment and −1.4% at the SSV moment. Comparatively, the high‐temperature downstream region constructed by the PTD design weakens its negative impact as humidity increases. For ∆T = 20 °C, compared to ∆T = 0 °C, the decrease in ξ downstream is reduced from 11% at TVM and 9.4% at SSV when RH = 35%, gradually decreasing to 10.9% at TVM and 6% at SSV when RH = 60%, and finally reaching 2% and 0.7% at TVM and SSV, respectively, when RH = 100%. Overall, the increase in humidity gradually weakens the positive effect of the PTD on the upstream region, while reducing its negative impact on the downstream region. The alleviation of the negative effects on the downstream increases with the rise in humidity, but it is still insufficient to completely counterbalance the weakening effect on the upstream region. However, in practical applications, the PTD design may lead to a more significant enhancement in HCD dynamic response performance, as higher humidity could cause more pronounced flooding downstream as the active area increases.

In contrast, for the NTD design, as RH increases from 35% to 60% and then to 100%, the negative impact on the average local current density in the upstream region gradually weakens. For ∆T = ‐20 °C, relative to ∆T = 0 °C, the reduction in ξ upstream decreases from 15.3% at the TVM moment and 8.9% at the SSV moment when RH = 35%, to 4.9% and 5.6% at RH = 60%, and finally to 1.4% and 4.5% at RH = 100%. As humidity increases, the drying effect caused by the higher temperature in the upstream region of the NTD design gradually decreases, alleviating the suppression of the upstream power generation capacity due to membrane dehydration. However, the relatively low temperature created downstream by the NTD design gradually weakens its positive effect with increasing humidity, eventually turning into a negative impact. For example, with ∆T = −20 °C compared to ∆T = 0 °C, the enhancement in contribution rate ξ downstream decreases from 11.7% at TVM and 1.8% at SSV when RH = 35%, to −2% at TVM and 0.43% at SSV when RH = 60%, and finally to −7.6% at TVM and −2% at SSV when RH = 100%. The increased humidity raises the water content in the reactant gases, making the downstream region more susceptible to flooding at HCDs due to lower temperatures. In summary, as humidity increases, the positive effect of the NTD design on the downstream region gradually weakens, eventually turning negative under high humidity. Meanwhile, the negative impact on the upstream region diminishes with increasing humidity. However, the alleviation of the negative impact upstream is limited and cannot fully compensate for the overall detrimental effect of the NTD design on performance. The evolution of ξ elucidates the trend of performance degradation in the PTD design and the escalating adverse effects of the NTD design as humidity increases.

**Figure 9:**
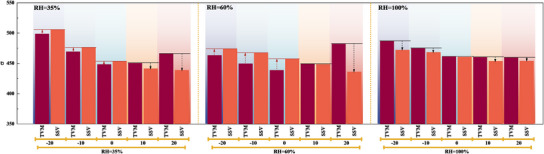
Quantitative comparison of the impact of different TD designs on the evolution of standard deviation σ during rapid HCD loading response process under different humidity conditions. (left: RH = 35%; middle: RH = 60%; right: RH = 100%).

**Figure** **9** compares the standard deviation σ evolution during response to the rapid HCD loading process under different humidity conditions and TD designs. Similarly, the TVM and SSV moments are taken as reference points, aiming to investigate the impact of TD designs on the uniformity of local current density distribution under different humidity conditions during the rapid loading response process. NTD and ZTD designs intensify the inhomogeneity of local current density distribution during the response period until stability is reached under RH = 35% and 60%. Both designs exacerbate the drying of the inlet region, causing the upstream power generation capacity to be consistently lower than that in the downstream region. The PTD design can effectively mitigate this issue under low humidity conditions. By lowering the upstream temperature and raising the downstream temperature, this design enhances the power generation capacity in the upstream region while moderately suppressing the power generation capacity in the downstream region, thereby improving the uniformity of local current density distribution across the entire cell. Moreover, when the reactant gases are fully humidified (RH = 100%), the PTD design still positively affects the standard deviation at both the TVM and SSV moments. This phenomenon occurs because the PTD design marginally reduces the power generation capacity in the upstream region, achieving a more balanced local current density distribution.

## 4 Conclusion 

This study introduces an innovative TD design featuring an in‐plane temperature gradient, which optimizes the temperature distribution in PEMFCs by reconfiguring the liquid cooling channels along the gas flow direction. This design substantially improves the dynamic response performance of PEMFCs under rapid HCD loading by optimizing temperature distribution, thereby enhancing water‐gas‐heat characteristics. Key moments include load initiation, TVM, and SSV to systematically investigate the effects and mechanisms of TD designs (including varying directions and magnitudes) on the dynamic performance of PEMFCs under different humidity conditions. The main conclusions are summarized as follows:

1. Under low humidity conditions, the PTD design enhances PEMFC HCD dynamic response performance by increasing TVM and SSV, alleviating VU, and reducing associated energy losses during the response. When RH = 35%, compared to the conventional ZTD design, the maximum increase in TVM is 18.2%, VU decreases by 12.5%, and SSV increases by 5.67%. Consequently, the electricity generation during the response process rises by 7%. In contrast, the NTD design exhibits adverse effects in all aspects.
2. Using EIS and local current density monitoring techniques, the influence of TD designs on dynamic response performance was comprehensively analyzed. The PTD design enhances upstream power generation by improving localized RH and achieves superior dynamic response performance and uniform current distribution. Conversely, the NTD design induces membrane dehydration upstream and flooding downstream, severely limiting voltage recovery and overall efficiency.
3. The effect of TD designs on rapid HCD loading performance under varying humidity conditions was investigated. The results show that as humidity increases, the negative impact of the NTD design persists, while the positive effect of the PTD design gradually diminishes. As the RH increases from 35% to 100%, the VU transitions from a positive parabolic to a “W” shape and ultimately to an inverted parabolic curve.
4. This study examined how increasing humidity alters the effect of TD designs on response performance. Higher RH intake causes upstream flooding under the PTD design, suppressing HCD reactions and diminishing its positive effects. The mitigation of downstream drying due to increased humidity is insufficient to compensate for upstream performance losses. Conversely, the negative impact of the NTD design intensifies with rising RH, driven by persistent upstream dryness and downstream flooding, further hindering HCD performance.

## Conflict Of Interest

The authors declare no conflict of interest.

## Author Contributions

Fengyang Cai: Writing–original draft, Shanshan Cai: Methodology, Zhengkai Tu: Supervision, Siew Hwa Chan: Resources

## Data Availability

The data that support the findings of this study are available from the corresponding author upon reasonable request.
